# A rare presentation of cardiac and hepatic hydatid cysts in a young female: a case report

**DOI:** 10.1093/jscr/rjae553

**Published:** 2024-08-28

**Authors:** Yerkhanat Khuanbai, Bagdat Alataev, Yermagambet Kuatbayev, Nurzhan Bikhanov

**Affiliations:** Clinical and Academic Department of Surgery, CF “University Medical Center”, Nazarbayev University, Astana 010000, Kazakhstan; Clinical and Academic Department of Surgery, CF “University Medical Center”, Nazarbayev University, Astana 010000, Kazakhstan; Department of Cardiac Surgery, National Research Cardiac Surgery Center, Astana 010000, Kazakhstan; Clinical and Academic Department of Surgery, CF “University Medical Center”, Nazarbayev University, Astana 010000, Kazakhstan

## Abstract

Hydatid disease is an endemic parasitic infection caused by the larval stages of the tapeworm *Echinococcus*. It is highly endemic in Kazakhstan, with both *Echinococcus granulosus* and *Echinococcus multilocularis* widely distributed throughout the country. Hydatid disease can affect almost any organ in the body, with the liver and lung being the most common sites, while cardiac involvement is rare. We report a case of a young female presenting with a 1-year history of stabbing precordial pain, shortness of breath, and weakness. She had a hydatid cyst involving the myocardium of the left ventricle and the left lobe of the liver. The patient underwent surgical resection and received albendazole medication as part of the treatment regimen.

## Introduction

Echinococcosis is a parasitic disease caused by the larval stages of tapeworms from the genus *Echinococcus* and leads to cyst formation in human organs. Dogs, hosting adult tapeworms, shed eggs in their feces, thus transmitting the infection to humans through contaminated food or water ingestion [1]. Echinococcosis is highly endemic in Kazakhstan, with both *Echinococcus granulosus* and *Echinococcus multilocularis* widely distributed throughout the country, and the incidence of human cystic echinococcosis is recorded at 5 cases per 100 000 people [2]. While hydatid cysts are most commonly found in the liver (50%–70% of cases) and the lungs (20%–30% of cases), cardiac involvement is uncommon, accounting for only 0.5%–2% of all reported cases of hydatidosis, even in areas where the disease is endemic [3, 4]. The ventricular myocardium is the predominant cardiac site affected by hydatid cysts, with subsequent involvement of the coronary arteries and sub-epicardium. Right ventricular hydatid cysts have a lower prevalence (10%) than left ventricular hydatid cysts (60%) [5]. Fatal complications, such as anaphylactic shock, may arise from cardiac hydatid cysts, especially if situated in the left ventricular outflow tract, or alternatively, pulmonary embolism may occur if the hydatid cyst is in the right ventricular outflow tract [6, 7]. Surgical removal is the primary treatment for echinococcosis, even in asymptomatic cases, to prevent cystic rupture and severe complications; albendazole serves as long-term treatment for inoperable cases. Complete cyst removal significantly impacts cardiac manifestation prognosis, with albendazole administered before and after surgery to reduce the 10% post-surgery occurrence rate of hydatid cysts [8]. In this case report, we discuss a case of a 24-year-old female experiencing symptoms, including stabbing pain in the precordial region, dyspnea, fatigue, and generalized weakness. Subsequent investigations confirmed the diagnosis of hydatid cysts affecting both the myocardium of the left ventricle and the left lobe of the liver.

## Case presentation

We came across a case of a 24-year-old female living in Kazakhstan, who presented with a 1-year history of stabbing precordial pain, shortness of breath, and general weakness. She had no history of hepatitis, tuberculosis, or weight loss. She denied having viral hepatitis, pulmonary tuberculosis, diabetes, hypertension, asthma, or any skin diseases. According to the patient, the above complaints worsened 3 months ago. She reported no history of previous surgical operations, injuries, or blood transfusions. She had not traveled to countries with significant epidemiological concerns, nor had she been in contact with infectious patients. However, it should be noted that she had contact with pets. Additionally, she had no history of allergies. On physical examination, there were no abnormal findings. Upon physical examination, the blood pressure was 125/77 mmHg, the temperature was 36.2°C, and the pulse rate was regular at 96 per minute. Examinations of the cardiovascular, pulmonary, neurological, and gastrointestinal systems revealed no abnormalities. A chest X-ray was conducted and revealed no abnormalities. Following thorough laboratory analysis, the enzyme-linked immunosorbent assay (ELISA) test exhibited positivity for Immunoglobulin G (IgG), alongside minor decreases in albumin, total protein, UREA, sodium, HGB, HCT, and MCV levels, and a minor elevation in GFR and C-reactive protein (CRP) levels. All these parameters returned to normal levels by postoperative Day 10, as shown in Table 1. The laboratory results of the patient’s admission day and postoperative days are outlined in Table 1.

**Table 1:** Laboratory findings of the patient and reference values.

**Laboratory values**	**Admission**	**POD3**	**POD6**	**POD10**	**Reference values**
Albumin	2.88	2.81		4.20	3.50–5.20 g/dl
Total protein	6.06	5.97		7.61	6.60–8.30 g/dl
Alanine aminotransferase	11.80	6.50			0.00–33.00 U/L
Aspartate aminotransferase	33.50	9.00		14.80	0.00–32.00 U/L
CRP	4.00	14.25	14.35	0.40	0.00–0.50 mg/dl
Direct bilirubin	0.19	0.15			0.00–0.20 mg/dl
Total bilirubin	0.41	0.26			0.00–1.20 mg/dl
UREA	15.60	13.30	10.80	19.60	16.60–48.50 mg/dl
BUN	7.28	6.20			6.00–20.00 mg/dl
Creatinine	0.44	0.33	0.38	0.54	0.51–0.95 mg/dl
GFR	170.55	227.41	197.48	137.78	88.00–146.00 ml/min
Potassium	3.80	3.80	3.80	5.30	3.50–5.10 mmol/L
Calcium ionized	1.22	1.23	1.24	1.30	1.00–1.15 mmol/L
Sodium	133.00	135.00	137.00	140.00	136.00–145.00 mmol/L
WBC	10.39	9.17	6.89	7.81	4.50–11.00 × 109/L
RBC	3.90	2.92	3.86	4.24	3.80–5.50 × 1012/L
HGB	94.00	71.00	98.00	118.00	115.00–140.00 g/L
HCT	30.50	23.20	31.00	35.70	35.00–45.00%
MCV	78.20	79.50	80.30	84.20	80.00–100.00
MCH	24.10	24.30	25.40	25.50	27.50–33.00
MCHC	30.80	30.60	31.60	30.30	31.00–38.00 g/dl
PLT	266.00	239.00	282.00	191.00	140.00–400.00 × 109/L
RDW-SD	47.50	50.70	51.20	57.80	36.40–46.30
RDW-CV	16.80	17.40	17.70	19.00	11.70–14.40%
PDW	8.10	8.30	8.50	9.20	9.00–17.00%
MPV	8.70	9.20	8.70	9.70	7.50–12.00
P-LCR	14.60	17.50	15.50	22.60	13.00–43.00
PCT	0.23	0.22	0.25	0.19	0.11–0.28
NEUT	9.46	6.42	3.73	4.24	1.56–6.13 × 103/μl
LYMPH	0.36	1.50	1.81	2.36	1.18–3.57 × 103/μl
MONO	0.56	0.79	0.73	0.56	0.24–0.82 × 103/μl
EO	0.00	0.41	0.56	0.60	0.04–0.54 × 103/μl
BASO	0.01	0.05	0.06	0.05	
IG#	0.03	0.02	0.02	0.08	0.00–7.00 × 103/μl
IG	0.30	0.20	0.30	1.00	0.00–72.00%
NEUT%	91.00	70.00	54.10	54.30	38.00–72.00%
LYMPH%	3.50	16.40	26.30	30.20	18.00–40.00%
MONO%	5.40	8.60	10.60	7.20	2.00–9.00%
EO%	0.00	4.50	8.10	7.70	0.00–5.00%
BASO%	0.10	0.50	0.90	0.60	0.00–1.00%
MicroR	10.80	10.50	9.80	7.20	0.30–3.90%
MacroR	3.50	2.90	3.50	4.70	2.90–4.80%
Fibrinogen	3.51				2.00–3.93 g/L

POD-Postoperative day.

**Figure 1:**
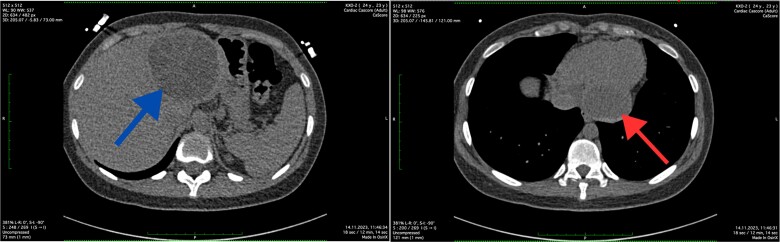
Axial CT scan showing the cystic lesion in the left lobe of the liver (blue arrow) and axial CT scan showing the cystic lesion in the left ventricle (red arrow).

The abdominal ultrasound revealed evidence of cyst formation in the left lobe of the liver, measuring ~10.0 × 8.0 cm in size. Additionally, a bend in the gallbladder neck was observed, accompanied by moderate bile stasis. On the transesophageal echocardiogram, no blood clots were detected in the left atrial appendage or cardiac cavities. The CT scan revealed uniform filling of the left portions of the heart with a contrast agent, indicating an absence of filling defects. Notably, structures within the myocardium near the diaphragm on the posterior lower wall of the left ventricle were identified as round-oval formations with clear contours. These formations exhibited densities ranging from +12 Hounsfield units to +44 Hounsfield units and measured 5.4 × 4.2 × 2.7 cm. Moreover, imaging of the left lobe of the liver depicted a heterogeneous structure attributed to the presence of a cystic formation. This formation displayed a density of up to +17 Hounsfield units and manifested as a round-oval structure with thickened walls. The cyst contained septa and areas of calcification, measuring up to 7.8 × 7.5 cm. Based on the CT scan findings, a diagnosis of echinococcal cyst was established. The presence of echinococcal cysts was noted in both the myocardium of the left ventricle of the heart and in the liver (Fig. 1).

**Figure 2:**
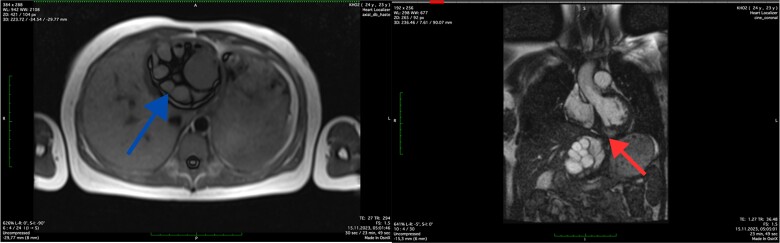
Axial MRI scan of the liver (blue arrow) and coronal MRI scan of the left ventricle (red arrow) showing the cystic lesions.

The MRI was conducted without contrast enhancement. Within the left ventricle, intramurally, specifically in the basal and medial segments (5, 6, 11, 12), a hyperintense MR signal was observed. These signals appeared round-oval in shape with clear, even contours and exhibited a heterogeneous structure due to internal partitions, measuring ~6.2 × 5.8 × 5.7 cm. The thoracic aorta displayed smooth contours, with a homogeneous MR signal reflecting the blood flow within its lumen. The trunk and branches of the pulmonary artery were identified and showed no signs of expansion. Additionally, in images captured in the left lobe of the liver, a multi-chamber formation was identified. This formation was round-oval in shape with smooth, clear contours and featured a thick capsule, measuring ~8.4 × 8.1 × 8.7 cm. The MRI findings indicated the presence of echinococcal cysts within the myocardium of the left ventricle and the left lobe of the liver (Fig. 2).

**Figure 3:**
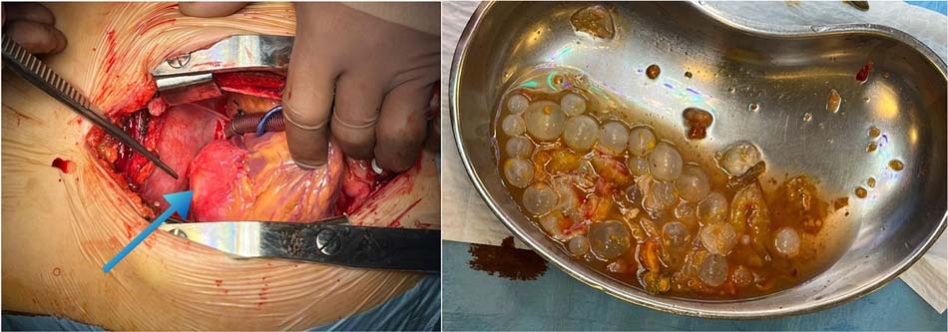
Perioperative image of cardiac hydatid disease showing the cystic lesion in the left ventricle (blue arrow) and ruptured hydatid cysts with undamaged daughter cysts.

Following a thorough review of the patient’s medical history, laboratory results, and imaging studies, a diagnosis of echinococcosis was confirmed. Specifically, echinococcal cysts were identified within the myocardium of the left ventricle and the left lobe of the liver. Subsequently, the patient underwent echinococcectomy procedures to remove cysts from both the myocardium of the left ventricle and the left lobe of the liver, receiving albendazole medication as part of the treatment regimen. The patient was administered total intravenous anesthesia and positioned supine. Following a median sternotomy, heparin was administered, and cardiopulmonary bypass (CPB) initiated. A 4 × 5 cm cyst was identified in the left ventricle, punctured, drained, and its membrane removed. The cavity was irrigated, and the patient was gradually warmed and weaned off CPB. Sternum osteosynthesis and chest closure were performed. Subsequent laparotomy revealed a voluminous liver cyst, which was excised. This combined approach minimized surgical risks and facilitated a coordinated recovery. Postoperative care included imaging and follow-ups, with an uneventful recovery period (Fig. 3). The patient had a smooth recovery and was discharged home 2 weeks after treatment, with instructions to continue taking albendazole 400 mg under the supervision of an infection specialist.

## Discussion

Cardiac hydatid cyst is mostly caused by myocardial invasion, which can arise from ruptured pulmonary echinococcal cysts in the pulmonary veins or from the coronary arteries [9]. Although echinococcal cysts can occur in different parts of the heart, the left ventricle is the most commonly affected; they frequently arise in the pericardium and myocardium layers; this increased involvement in the left ventricle could be explained by the left coronary artery’s predominance [10, 11]. Cardiac hydatid disease often accompanies hydatidosis affecting various organs. In our case report, the patient exhibited both cardiac and hepatic hydatid cysts. The clinical manifestations of cardiac hydatid disease vary from no symptoms to severe, life-threatening conditions. In our case report, cardiac hydatid disease manifested as sharp chest pain, difficulty breathing, and general weakness. Rupture of a cardiac hydatid cyst can lead to pulmonary embolism, acute stroke, cardiac tamponade, chest pain, valvular regurgitation, arrhythmias, and anaphylactic shock [12, 13]. Interventricular septal aneurysms, metastases, and heart malignancies are possible differential diagnoses to take into account. Treatment planning and diagnosis confirmation can be aided by the use of MRI and CT scans. The treatment of hydatid disease requires a multidisciplinary approach, typically coordinated in specialized medical centers where radiologists, surgeons, infectious disease specialists, and cardiologists collaborate. Surgical excision remains the cornerstone of treatment for cardiac hydatid disease, even in asymptomatic patients, due to the higher risk of hydatid cyst rupture and albendazole serves as long-term treatment for inoperable cases [14].

## Conclusions

The occurrence of cardiac hydatid cyst is uncommon, and differentiating it from other cardiac conditions poses a clinical difficulty. Particularly in regions where the disease is prevalent, such as Kazakhstan, clinicians should be vigilant regarding the possibility of cardiac hydatid disease affecting the heart upon encountering a cardiac cyst.

## Disclosures

Informed consent: Written informed consent was obtained from the patient for the publication of the patient's anonymized information in this case report.

Conflicts of interest: In compliance with the ICMJE uniform disclosure form, all authors declare the following: Payment/services info: All authors have declared that no financial support was received from any organization for the submitted work.

Financial relationships: All authors have declared that they have no financial relationships at present or within the previous three years with any organizations that might have an interest in the submitted work.

Other relationships: All authors have declared that there are no other relationships or activities that could appear to have influenced the submitted work.

## References

[1] McManus DP , Zhang W, Li J, et al. Echinococcosis. Lancet 2003;362:1295–304. 10.1016/S0140-6736(03)14573-4. 14575976

[2] Abdybekova A , Sultanov A, Karatayev B, et al. Epidemiology of echinococcosis in Kazakhstan: an update. J Helminthol 2015;89:647–50. 10.1017/S0022149X15000425. 26160276

[3] Dursun M , Terzibasioglu E, Yilmaz R, et al. Cardiac hydatid disease: CT and MRI findings. AJR Am J Roentgenol 2008;190:226–32. 10.2214/AJR.07.2035. 18094316

[4] Sensoz Y , Ozkokeli M, Ates M, et al. Right ventricle hydatid cyst requiring tricuspid valve excision. Int J Cardiol 2005;101:339–41. 10.1016/j.ijcard.2004.01.043. 15882692

[5] L’aarje A , Lyazidi S, Kitane Y, et al. Cardiac hydatid cyst of the right ventricle: severe localization. J Cardiol Cases 2017;16:138–40. 10.1016/j.jccase.2017.06.009. 30279818 PMC6149277

[6] Orhan G , Bastopcu M, Aydemir B, et al. Intracardiac and pulmonary artery hydatidosis causing thromboembolic pulmonary hypertension. Eur J Cardiothorac Surg 2018;53:689–90. 10.1093/ejcts/ezx330. 28958014

[7] de Gregorio C, Ferrazzo G, Ceresa F, et al. Dynamic right ventricular outflow tract obstruction by cardiac hydatic cysts: a multimodality imaging study. J Clin Ultrasound 2021;49:690–2. 10.1002/jcu.22993. 33634879

[8] Moro P , Schantz PM. Echinococcosis: a review. Int J Infect Dis 2009;13:125–33. 10.1016/j.ijid.2008.03.037. 18938096

[9] Banisefid E , Baghernezhad K, Beheshti R, et al. Cardiac hydatid disease; a systematic review. BMC Infect Dis 2023;23:600. 10.1186/s12879-023-08576-3. 37705012 PMC10500901

[10] Tufekcioglu O , Birincioglu CL, Arda K, et al. Echocardiography findings in 16 cases of cardiac echinococcosis: proposal for a new classification system. J Am Soc Echocardiogr 2007;20:895–904. 10.1016/j.echo.2006.12.012. 17617317

[11] Firouzi A , Neshati Pir Borj M, Alizadeh GA. Cardiac hydatid cyst: a rare presentation of echinococcal infection. J Cardiovasc Thorac Res 2019;11:75–7. 10.15171/jcvtr.2019.13. 31024677 PMC6477106

[12] Leila A , Laroussi L, Abdennadher M, et al. A cardiac hydatid cyst underlying pulmonary embolism: a case report. Pan Afr Med J 2011;8:12. 10.4314/pamj.v8i1.71061. 22121421 PMC3201579

[13] Malamou-Mitsi V , Pappa L, Vougiouklakis T, et al. Sudden death due to an unrecognized cardiac hydatid cyst. J Forensic Sci 2002;47:1062–4. 10.1520/JFS15520J. 12353547

[14] Brunetti E , Kern P, Vuitton DA. Writing panel for the WHO-IWGE: expert consensus for the diagnosis and treatment of cystic and alveolar echinococcosis in humans. Acta Trop 2010;114:1–16. 10.1016/j.actatropica.2009.11.001. 19931502

